# How Is the Lung Cancer Incidence Rate Associated with Environmental Risks? Machine-Learning-Based Modeling and Benchmarking

**DOI:** 10.3390/ijerph19148445

**Published:** 2022-07-11

**Authors:** Kung-Min Wang, Kun-Huang Chen, Chrestella Ayu Hernanda, Shih-Hsien Tseng, Kung-Jeng Wang

**Affiliations:** 1 Department of Industrial Management, National Taiwan University of Science and Technology, Taipei 106, Taiwan; albert.hua@msa.hinet.net (K.-M.W.); m10901847@mail.ntust.edu.tw (C.A.H.); shtseng@mail.ntust.edu.tw (S.-H.T.); 2 College of Management and Design, Ming-Chi University of Technology, Taipei 243, Taiwan; kunhuang@mail.mcut.edu.tw

**Keywords:** lung cancer incidence rate, predictive model, machine learning algorithm, cubist model tree, random forest, feature selection, variable importance

## Abstract

The lung cancer threat has become a critical issue for public health. Research has been devoted to its clinical study but only a few studies have addressed the issue from a holistic perspective that included social, economic, and environmental dimensions. Therefore, in this study, risk factors or features, such as air pollution, tobacco use, socioeconomic status, employment status, marital status, and environment, were comprehensively considered when constructing a predictive model. These risk factors were analyzed and selected using stepwise regression and the variance inflation factor to eliminate the possibility of multicollinearity. To build efficient and informative prediction models of lung cancer incidence rates, several machine learning algorithms with cross-validation were adopted, namely, linear regression, support vector regression, random forest, K-nearest neighbor, and cubist model tree. A case study in Taiwan showed that the cubist model tree with feature selection was the best model with an RMSE of 3.310 and an R-squared of 0.960. Through these predictive models, we also found that apart from smoking, the average NO_2_ concentration, employment percentage, and number of factories were also important factors that had significant impacts on the incidence of lung cancer. In addition, the random forest model without feature selection and with feature selection could support the interpretation of the most contributing variables. The predictive model proposed in the present study can help to precisely analyze and estimate lung cancer incidence rates so that effective preventative measures can be developed. Furthermore, the risk factors involved in the predictive model can help with the future analysis of lung cancer incidence rates from a holistic perspective.

## 1. Introduction

An estimated 19.3 million new cancer cases occur worldwide each year and result in nearly 10.0 million people dying from the disease [1,2]. Lung cancer accounted for 1.80 million deaths or about 18% of the total cancer deaths in 2020. Many variables, including genetic predisposition, unhealthy diet, environmental exposure, and air pollution, may influence lung cancer occurrences separately or in combination with tobacco smoking [3,4].

By far the most prevalent cause of lung cancer mortality is smoking, accounting for around 80% of all lung cancer fatalities globally. In addition to smoking, experts believe that air pollution is also one of the major contributors to lung cancer incidence. Traffic emissions, industrial pollutants, coal combustion, steel production, and suspended road dust are the primary contributors to air pollution. Particulate matter 2.5 (PM_2.5_) is the most harmful group of pollutants to a person’s health, followed by ozone and nitrogen oxides [5,6]. Coleman et al. [7] concluded that exposure to PM_2.5_ contributes to lung cancer mortality and may be a risk factor for other types of cancer. According to Hvidtfeldt et al. [8], long-term exposure to ambient PM_2.5_ is related to lung cancer, even at concentrations below the current EU limit levels and perhaps the WHO Air Quality Guidelines. Kim et al. [9] found that exposure to the primary air pollutants (PM_2.5_, PM_10_, and NO_2_) is related to an elevated risk of cancer death across the board [10], including lung cancer. Other factors, such as socioeconomic status, employment status, marital status, and living environment, are also linked with the occurrence of lung cancer.

The recording of a disease mortality rate serves as statistical data that is used to monitor the causes of death and life expectancy, and allows for the determination of developmental policies in an area. Mortality rate data is also closely related to incidence rate data. As for analyzing the mortality rate of a disease, the first step involves analyzing the incidence rate of the disease itself. Therefore, generating a prediction model to analyze the incidence rate of a disease is necessary. Rahib et al. [11] used population growth and cancer trends to estimate cancer incidences in the USA. According to the findings, leading cancer rates and fatalities in the United States will be significantly different in 2040 than they are now. Jakobsen et al. [12] projected the future lung cancer occurrence, death, and prevalence in Denmark. For the years 2016 to 2030, a forecast of future numbers of yearly incident cases, fatalities, and resulting prevalent case numbers was developed using the concepts of a “stock and flow” model for a closed population. The data suggest that lung cancer is being detected at an earlier stage, that the incidence will plateau, that death will decline further, and that the prevalence will continue to rise significantly.

Machine learning algorithms have been adopted to predict the incidence rate, mortality rate, or survivability of cancer [13]. Sekeroglu and Tuncal [14] used linear regression (LR), support vector regression (SVR), decision trees, long short-term memory neural networks (NN), backpropagation NN, and radial basis function NN to build cancer incidence rate prediction models for the European continent. They found that LR and SVR outperformed the other models with R-squared values of 0.99 and 0.98, respectively. Tuncal et al. [15], proposed several machine learning algorithms, including SVR, backpropagation NN, and long short-term memory NN, to provide an effective and rapid prediction of lung cancer incidence. The results show that SVR gives better results than the other considered algorithms.

Studies on lung cancer incidence rates were devoted to clinical studies but only a few addressed this issue from a holistic perspective of the social, economic, and environmental dimensions. Therefore, in this study, we aimed to build prediction models for the incidence rate of lung cancer in the whole country of Taiwan using machine learning algorithms by considering several risk factors or features for lung cancer, such as air pollution, tobacco use, socioeconomic status, employment status, marital status, and living environment. These risk factors or features were comprehensively surveyed.

## 2. Materials and Method

### 2.1. Data Source

The Ministry of Health and Welfare (MOHW) statistics revealed that the cancer death rate in Taiwan in 2020 fell slightly for the first time since 2009; however, cancer was still the biggest cause of mortality in Taiwan that year, claiming more than 50,000 lives [16,17]. Lung cancer is also one of the most frequently diagnosed cancers in Taiwan [18,19,20]. The incidence of lung cancer is growing rapidly, making Taiwan ranked 15th in the world and 2nd in Asia for the incidence of lung cancer [21].

**Figure 1:**
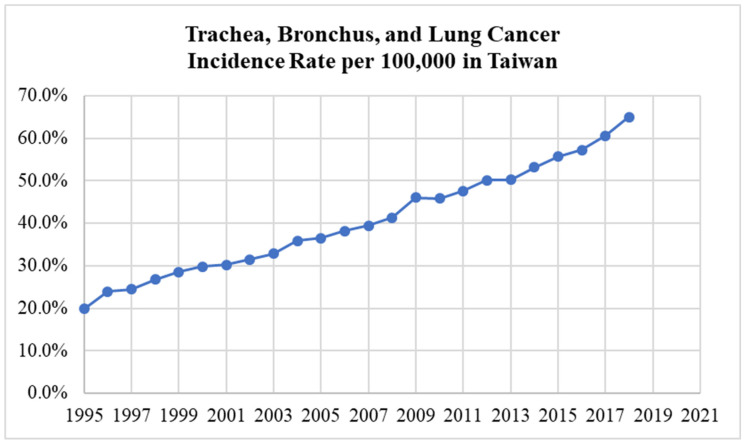
The trachea, bronchus, and lung cancer incidence rate in Taiwan.

The lung cancer incidence rate dataset from 1995 to 2018 used in this study was obtained from the Cancer Registry Report by the Health Promotion Administration, Taiwan [18,19]. The HPA is a government organization that was formed to be responsible for health promotion and the prevention of non-communicable diseases. The organization is also responsible for conducting public health surveillance and related research, as well as dealing with other specialized health topics. Figure 1 shows the graph of the trachea, bronchus, and lung cancer incidence rates per 100,000 in the whole country of Taiwan from 1995 to 2018, which grew more than threefold over this period.

Other datasets, including air pollution, tobacco use, socioeconomic status, employment status, marital status, and living environment data [22], were also used in this study. The air pollution dataset (such as carbon monoxide, nitrogen dioxide, sulfur dioxide, ozone, and particulate matter 10) was obtained from the Air Quality Annual Report by Environmental Protection Administration (EPA), Taiwan [5,6]. To improve data exchange and information services, the EPA created a hierarchical air quality monitoring system to thoroughly integrate real-time monitoring data given by air monitoring stations of various authorities. The dataset for each type of air pollution for the whole country of Taiwan was obtained by averaging the data from a total of 22 administrative divisions of Taiwan provided by the EPA. Registered vehicle data was obtained from the Annual Transportation Report, Ministry of Transportation and Communication (MOTC), Taiwan [23,24]. The MOTC is tasked with regulating all aspects of transportation and communication, with the MOTC statistics section being in charge of gathering, analyzing, and disseminating data on Taiwan’s transportation and communications industry.

Industry-related data from the Factory Operation Census Report, Ministry of Economic Affairs (MOEA), Taiwan, were also included in the air pollution dataset used in this study. This annual report was released by the MOEA statistics department, which is responsible for developing statistical sets to show economic changes due to the impacts of changes in industry, trade, and services, as well as the future development of economic activity [25,26,27]. Tobacco use, socioeconomic status, employment status, marital status, and living environment data were obtained from the National Statistics of the Directorate-General of Budget, Accounting and Statistics (DGBAS), Taiwan. The Executive Yuan’s DGBAS handles national budgetary, accounting, and statistical affairs which complement each other in an integrated system [28]. The tobacco use dataset included tobacco consumption per capita aged 18 and over (pieces/year), and the percentage of smokers from the population aged 18 and over. The employment status dataset included the percentage of employed from the civilian population aged 15 and over, as well as the unemployment rate. Registered workers from all occupations (such as mining and quarrying, manufacturing, electricity and gas supply, water supply, and service providers) were included in this percentage of employed. The aim was to find out whether people who had jobs affected the incidence of lung cancer, whether it was due to the work environment or work stress.

The above-mentioned datasets from 1995 to 2020 with a total of 26 years (the sample size) for each variable were collectively used in building a machine learning model in this study. Then, the imputation method was applied to fill in missing feature values with a reasonable approximated value based on the existing feature values. The most frequent method of imputation is to replace missing values in a feature with a measure of that feature’s central tendency [12,29]. The mean or median is most usually employed for continuous features, whereas for categorical features, the mode is most commonly used. When the data is skewed, utilizing the median value to replace missing values and provide robustness is recommended [30]. Therefore, in this study, the median of each variable was used to replace missing values. 

### 2.2. Variables

The independent variable (predictor) is one of the important components in building a machine learning model. In this study, various independent variables were used to build a predictive model of the lung cancer incidence rate in Taiwan (dependent variable). Several studies showed that air pollution appears to slightly increase the risk of lung cancer. Therefore, we considered various air pollutants as independent variables in this study, as well as the number of registered vehicles and the number of factories that contribute to air pollution. Tobacco use or smoking is also one of the factors that cannot be separated when discussing lung cancer, where smoking itself is associated with various other risk factors for lung cancer.

Several studies linked socioeconomic status (SES) to lung cancer, with those from lower socioeconomic backgrounds having the greatest incidence rates [31]. Tobacco use was responsible for 11.7% of the entire sickness burden in the lowest socioeconomic regions (individuals who are most socioeconomically disadvantaged), whereas it was only 6.5% in the highest socioeconomic regions (those who experienced the least disadvantage). After age was taken into account, the same statistics demonstrated that the burden of illness caused by tobacco smoking was 2.6 times higher in the lowest socioeconomic regions than in the highest socioeconomic regions [32]. According to the National Drug Strategy Household Survey [33], daily smoking is still more common among those in poor socioeconomic groups, people who live in distant or remote places, and people who are unable to work or are jobless. De Vogli and Santinello [34] also analyzed the link between smoking and unemployment, finding that jobless people were 2.78 times (95% confidence interval (CI) 1.68 to 4.62) more likely to smoke than managers and professionals after adjusting for higher demographic characteristics.

Marital status is also commonly linked to lung cancer survival, while research on the particular association between the two produced inconsistent results. Tannenbaum et al. [35] concluded that lung cancer patients who are married or widowed have a better prognosis than those who are never married or who are divorced. On the other hand, another study discovered that marriage was not a significant predictor of survival [36]. Therefore, we considered the marital status factor in this study to determine whether there was an association with the incidence of lung cancer. In addition, we also considered the living environment data, namely, the rate of people living in one-story buildings, rate of people living in apartments six stories high or over, rate of days with a PSI of more than 100, availability rate of public sanitary sewers, rate of heavy-polluted sections, rate of unqualified drinking water, and rate of proper disposal. According to the US Environmental Protection Agency (EPA), radon is the second leading cause of lung cancer in nonsmokers and the top in smokers. Indoors (homes and other buildings) may have high levels of radon, especially in basements. People in areas with unqualified drinking water (such as high arsenic levels) have a greater risk of lung cancer too. 

Finally, a total of 20 risk factors (independent variables) used as considerations in building predictive models of lung cancer incidence rate in Taiwan (dependent variable) are shown in Table 1.

### 2.3. Method

**Figure 2:**
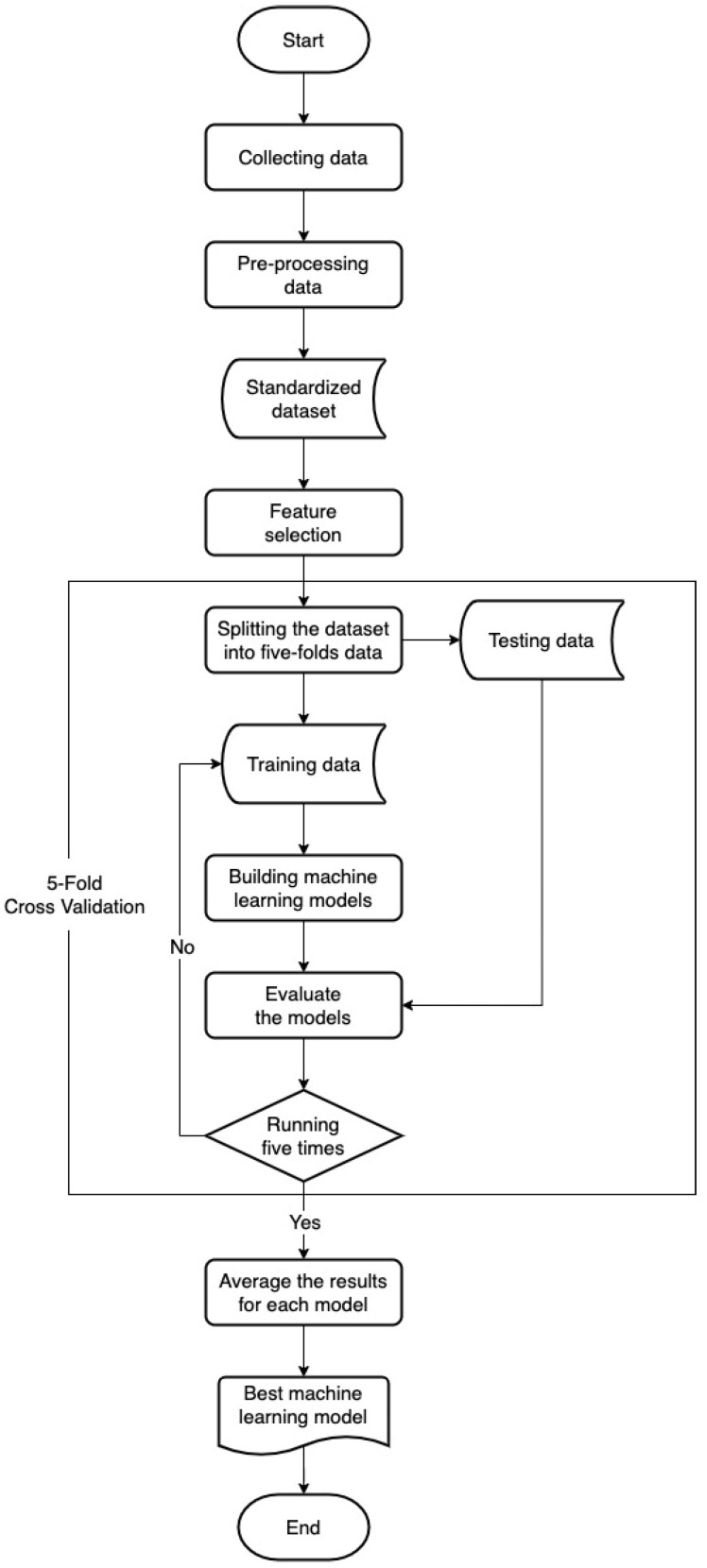
Research process.

The study procedure is shown in Figure 2. Collecting the required data was carried out as the first step in this study, then data pre-processing was carried out to fill in the missing data and standardize the data. Data standardization is known as the process of converting data into a common format that permits individuals to explore, evaluate, and make use of it. The term “standardization” refers to the act of placing a variety of variables on a single scale so that scores can be compared. Data standardization has the ability to eliminate data utilization roadblocks, such as metadata uncertainties, data transformation challenges, and missing data. Data transformation has multiple challenges, such as merging data from various time intervals into a cohesive data collection. Other obstacles may occur as a result of the requirement to rearrange data into new datasets that are integrated with different internal structures. These obstacles make data integration harder, which can lead to higher costs. This procedure can eliminate at least some of these roadblocks, resulting in improved data flow and machine learning [37,38,39]. Then, before building a predictive model, feature selection was carried out to reduce the number of input variables by selecting influential features (optimal features) and overriding features that had no effect.

In this study, we used five supervised learning algorithms to build a predictive model, namely, linear regression, SVR, random forest, K-nearest neighbor, and cubist model tree. Details on these algorithms are found in Appendix A. The R programming language was used to build prediction models. Parameters for each of these machine learning algorithms were mostly set to random value combinations generated by the Caret package in R. For each algorithm, parameter tuning was automatically performed using tuneLength, which is one of the built-in capabilities of the Caret package and implements a cross-validation grid search approach. In this study, we used tuneLength = 10, which denotes 10 random tuning parameter combinations to try for each tuning parameter. To evaluate the predictive models obtained from each algorithm, 5-fold cross-validation was used to avoid over-fitting. The RMSE and R-squared results from each fold were then averaged and compared to determine which algorithm was the best at building a predictive model of lung cancer incidence rate in Taiwan.

### 2.4. Feature Selection

In building a predictive model, we considered the multicollinearity issue where the independent variables are correlated with each other. Multicollinearity causes the estimator to have a large variance, and as a result, the estimation interval tends to be larger such that the independent variable is not statistically significant, even though the coefficient of determination (R-squared) is high, making it difficult to obtain an accurate estimate [10,40,41]. This condition is often referred to as overfitting, which is the main concern during feature selection and it must be ensured that it does not occur. A very high correlation between independent variables results in regression model estimators that are biased, unstable, and may be far from their predicted values [42].

The variance inflation factor (VIF), which quantifies how much the variance of a predicted regression coefficient increases when predictors are linked, is one technique to determine multicollinearity [43,44,45,46]. When orthogonal independent variables are linked linearly, VIF is a factor that indicates how much the variance of the regression estimator coefficient increases when compared with the orthogonal independent variables. A VIF value greater than 10 can be used as a strong indicator of multicollinearity. Other criteria were proposed, such as predictors with VIF values greater than 5 potentially significantly contributing to multicollinearity and requiring more investigation [47]. Standard errors for one or more individual partial regression coefficients might be excessively exaggerated when several of the predictors are engaged in significant linear correlations among themselves. In the setting of other explanatory factors, this tends to result in conclusions of a probable lack of distinctive significance for substantively relevant regressors [44]. As a technique for measuring probable (near) multicollinearity, the VIF equation is defined as given in Equation (1).

(1) $$V_{j}=1/\left(1-R_{j}^{2}\right)$$

where $R_{j}^{2}$ represents the *R*^2^ index when the *j*th explanatory variable is regressed on the remaining independent variables *j* = 1, …, k [46].

This research had 20 independent variables, some of which had sufficient underlying data to accurately predict the outcome. However, this set of predictors might include non-informative factors, which could have an influence on the performance. After the stepwise regression procedure, a feature selection strategy was used to limit the predictor set to a smaller set that only contained the useful predictors. A VIF value greater than 5 was used as an indicator of multicollinearity.

### 2.5. Evaluation Criteria

After building a machine learning model, the model evaluation was undertaken by using the testing data. K-fold cross-validation is generally used to evaluate the performance of machine learning models, especially on a limited dataset. In this study, 5-fold cross-validation was used to reduce the bias that might be caused by random sampling. The dataset was initially separated into five random disjoint folds with approximately equal numbers of occurrences. Then, one by one, each fold took on the duty of testing the model created by the other 4 (k−1) folds. Because the partition was random, the variation in the accuracy estimates for statistical inference might be rather high. In assessing the fit of the regression, two statistic values were used, namely, the root-mean-square error (RMSE) and R-squared. RMSE is the square root variance of the residuals, which indicates the absolute fit of the predictive model to the observed data. RMSE may be defined as the standard deviation of the unexplained variance and has the advantage of being in the same units as the response variable. If the model’s primary goal is prediction, the RMSE is the most essential fit criteria and a good indicator of how well it predicts the response [48,49]. The RMSE equation can be seen as Equation (2).

(2) $$\mathrm{RMSE}=\sqrt{\frac{\sum^{\;}{\left(A_{t}-F_{t}\right)}^{2}}{n}}$$

where *A_t_* is the current value in period *t*, *F_t_* denotes the projected value in period t, and *n* denotes the number of periods utilized in the computation [50]. This RMSE value is a suitable measure of accuracy for comparing prediction errors of different models or model configurations for a given variable, with a smaller RMSE value (close to 0) indicating that the prediction results are more accurate.

R-squared is a measure of how much the interaction of independent factors influences the value of the dependent variable. R-squared has the benefit of a straightforward scale that ranges from 0 to 1. The value of R-squared increases proportionally when the regression model is improved [48]. 

## 3. Results and Discussion

### 3.1. Key Features of Lung Cancer Incidence

**Figure 3:**
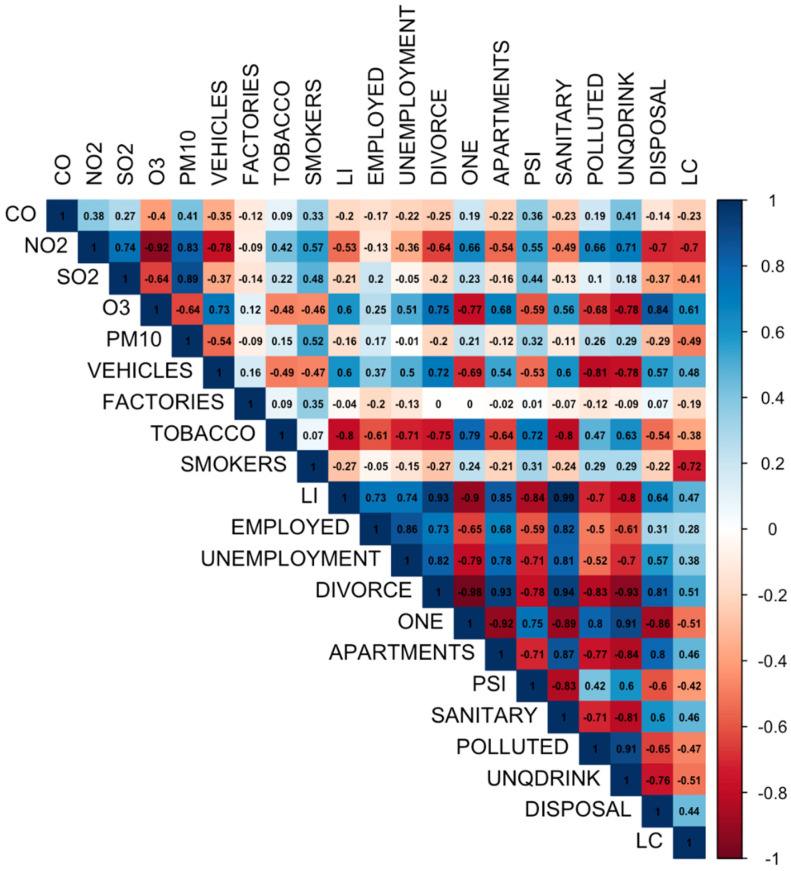
Correlation plot.

Twenty predictor variables that have an association with the incidence of lung cancer were considered when constructing our predictive model. The correlation plot of these 20 predictor variables with the dependent variable of lung cancer incidence rate (LC) can be seen in Figure 3. Before building a predictive model, stepwise regression was performed to analyze all considered predictor variables so that a useful subset of predictors could be identified. Stepwise regression itself is a method that is used to obtain the best model from a regression analysis. Then, to ensure that there is no multicollinearity, feature selection is carried out by calculating the VIF value of each variable in the subset of useful predictors from the stepwise regression results. In this study, predictor variables with a VIF value of more than 5 were eliminated. The subset of predictors from the stepwise regression consisted of 15 predictor variables, while the subset of predictors from the feature selection based on the VIF value consisted of 8 predictor variables. These two subsets of predictors can be seen in Table 2 and were used to build the machine learning models.

The results indicated that eight key variables (NO_2_ concentration, number of registered vehicles, number of factories, tobacco consumption, percentage of smokers, percentage of employed, percentage of days measured with PSI more than 100, and percentage of proper refuse disposal) were included in the selected features based on the VIF value. 

On the other hand, the O_3_ concentration, PM_10_ concentration, unemployment rate, percentage of the population with divorce status, and number of households living in a one-story building were eliminated in the stepwise regression stage, which showed that these variables were not significantly associated with the dependent variable of lung cancer incidence rate. 

These statements are in line with the results of several studies [51,52] that showed significant associations between NO_2_ and NO_x_ concentrations and lung cancer risk. Vehicles are a major source of particulate matter, nitrogen oxides, carbon monoxide, and other pollutants, which contribute significantly to air pollution. According to a 2013 assessment by WHO’s International Agency for Research on Cancer (IARC), outdoor air pollution is carcinogenic to humans and is related to an increased incidence of cancer, particularly lung cancer [53]. Chen et al. [51] concluded that lung cancer risk rises significantly when people are exposed to traffic-related air pollution. Extensive research data also established smoking as a major cause of lung cancer and environmental tobacco smoke is regarded as a probable occupational carcinogen [33,54]. Smokers have a thirty-fold increased risk compared with nonsmokers of developing cancer [55]. Moon et al. [56] found that lung cancer incidence was higher in smokers but there was no increased risk of lung cancer with higher PM_10_ exposure.

The remaining variables (CO concentration, SO_2_ concentration, percentage of low-income persons, number of households living in apartments, percentage of public sanitary sewers, percentage of heavy-polluted sections, and percentage of unqualified drinking water) were only included in the stepwise regression stage. This showed that these variables had high VIF values (greater than 10), meaning that there were associated independent variables that were highly collinear with the other independent variables in the model (multicollinearity). Therefore, these remaining variables were removed at the feature selection stage. As a result, two sets of predictors could be obtained, namely, the predictor set from the stepwise regression and the predictor set from feature selection based on the VIF value. The predictor set from the stepwise regression was used to build predictive models, which were then referred to as models without feature selection. The prediction models that were generated using the predictor set from feature selection were then referred to as models with feature selection. Furthermore, the performance of each model was compared to determine whether eliminating multicollinearity could reduce the error of a model in this study.

### 3.2. Benchmarking of Machine Learning Algorithms

Table 3 shows the performance of the models without feature selection and with feature selection. It could be concluded that all machine learning models with feature selection in this study were models with strong effect sizes since their R-squared values were more than 0.7 [57]. This meant that each machine learning model with feature selection could explain the variation in the dependent variable well and the model fit the observed data. For machine learning models without feature selection, all models in this study except the linear regression model also had an R-squared value of more than 0.7, and thus, it can be said that the models had strong effect sizes. 

However, to determine the best machine learning model, it was necessary to consider the stability of the model’s performance at each fold. The fairly large R-squared range between folds in some models (e.g., the linear regression without feature selection) indicated that the performance of the model was not constant, meaning that the algorithms were not able to build a good predictive model for the data used. Table 3 indicates that the cubist model tree with feature selection gave the lowest mean RMSE value when predicting the incidence rate of lung cancer. The RMSE average value of the fivefold cross-validation using the cubist model tree with feature selection was 3.310 with an R-squared of 0.960, meaning that 96.0% of the variation in the dependent variable (lung cancer incidence rate) could be explained by the model. In addition, the cubist model tree with feature selection had a fairly constant R-squared value for each fold; therefore, it could be concluded that feature selection was able to provide a predictive model with a lower error value.

### 3.3. Discussion

A metric, such as the accuracy of prediction, cannot fully describe the majority of real-world tasks, and thus, raises the need for interpretability [58]. Interpretability itself is the extent to which we can understand the explanation for a decision [59]. In addition to knowing that a predictive model performs well, knowing why a decision or prediction was made can help us to learn more about the problem, the data, and the reasons why a model might fail [60]. However, not all models can be interpreted easily, such as a “black box” model, which is a term for a model that is complex enough that it cannot be interpreted directly. Difficulties in understanding and interpreting a “black box” model can undermine confidence in the model and limit its use in certain fields, including health and medicine [16,61]. The random forest model is one of the “black box” models in which information about the relationships between model variables and outputs is hidden in the model structure [62].

A random forest model comprises a huge number of deep trees, each of which is trained on bagged data using random feature selection, making it hard to examine each tree individually to acquire a complete understanding of the random forest model’s decision process. One approach to interpreting and gaining insight into a “black box” model is to calculate the variable importance that represents the statistical significance of each variable used in relation to its effect on the resulting model. Quantifying the importance of features in a machine learning model helps to understand the global contribution of each feature to a model’s predictions.

**Figure 4:**
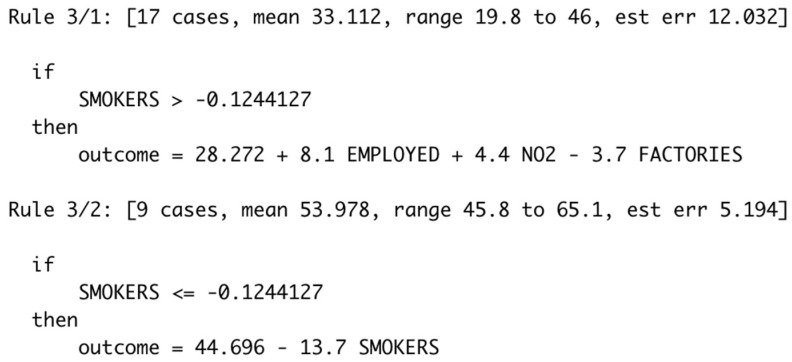
Cubist model tree with feature selection.

The results of this study showed that the cubist model with feature selection (RMSE: 3.310, R-squared: 0.960) was the best model for predicting the incidence rate of lung cancer in this study, followed by the random forest model without feature selection (RMSE: 4.837, R-squared: 0.895) and the random forest model with feature selection (RMSE: 4.922, R-squared: 0.894). These three models showed fairly constant performance results for each fold, indicating that the machine learning algorithms were not able to build good predictive models from the data used. The following cubist model tree with feature selection generated in this study can be considered when predicting the lung cancer incidence rate in Taiwan using standardized data (Figure 4).

The cubist model tree with feature selection above consists of two rules, where the first rule gives a greater contribution than the other rule to the accuracy of the model on the training data. When all conditions in a rule are met, the linear model in that rule is used to calculate the prediction of the lung cancer incidence rate. The first rule of the above model can be interpreted as saying that among all training cases, there were 17 cases that satisfied the condition of a standardized smokers rate greater than −0.124 and their lung cancer incidence rates ranged from 19.8 to 46 with an average value of 33.112. The model discovered that the target outcome value of these or other cases satisfying the condition could be modeled using the linear model formula in the first rule with an estimated error (est err) of 12.032.

Furthermore, it can be seen that “SMOKERS” (percentage of smokers), “NO_2_” (average NO_2_ concentration), “EMPLOYED” (percentage of employed), and “FACTORIES” (number of factories) variables contribute to the model, meaning that we could consider these variables as important risk factors of lung cancer incidence in Taiwan. The contribution of the “SMOKERS” (percentage of smokers) variable to the cubist model tree with feature selection was in line with the general public knowledge and, therefore, supported the reliability of this model. The World Health Organization highlights that tobacco smoking is the most common cause of lung cancer, accounting for more than two-thirds of all lung cancer deaths worldwide [63]. Quitting smoking can reduce the risk of lung cancer to about half of a smoker’s risk after 10 years of quitting smoking, and about 90% of lung cancers can be avoided by eliminating tobacco use [64].

Regarding the association between lung cancer and NO_2_ exposure, Hamra et al. [65] collected 20 relevant studies for analysis and the results showed that for every 10 μg/m^3^ increase in NO_2_ exposure, the risk of lung cancer increased by 4% (95% CI: 1%, 8%). One of the sources of NO_2_ is vehicles, where large diesel vehicles as mobile pollution sources are one of the main sources of NO_2_. Apart from NO_2_, vehicles also produce other pollutants, such as particulate matter, nitrogen oxides, and carbon monoxide. Therefore, exposure to high doses of NO_2_ and other pollutants may occur in areas with high traffic flow. Some other interesting variables, namely, the “EMPLOYED” (percentage of employed) variable and the “FACTORIES” (number of factories) variable can be related to the incidence of lung cancer via exposure in the work environment [1,25,66] or work stress [67].

**Figure 5:**
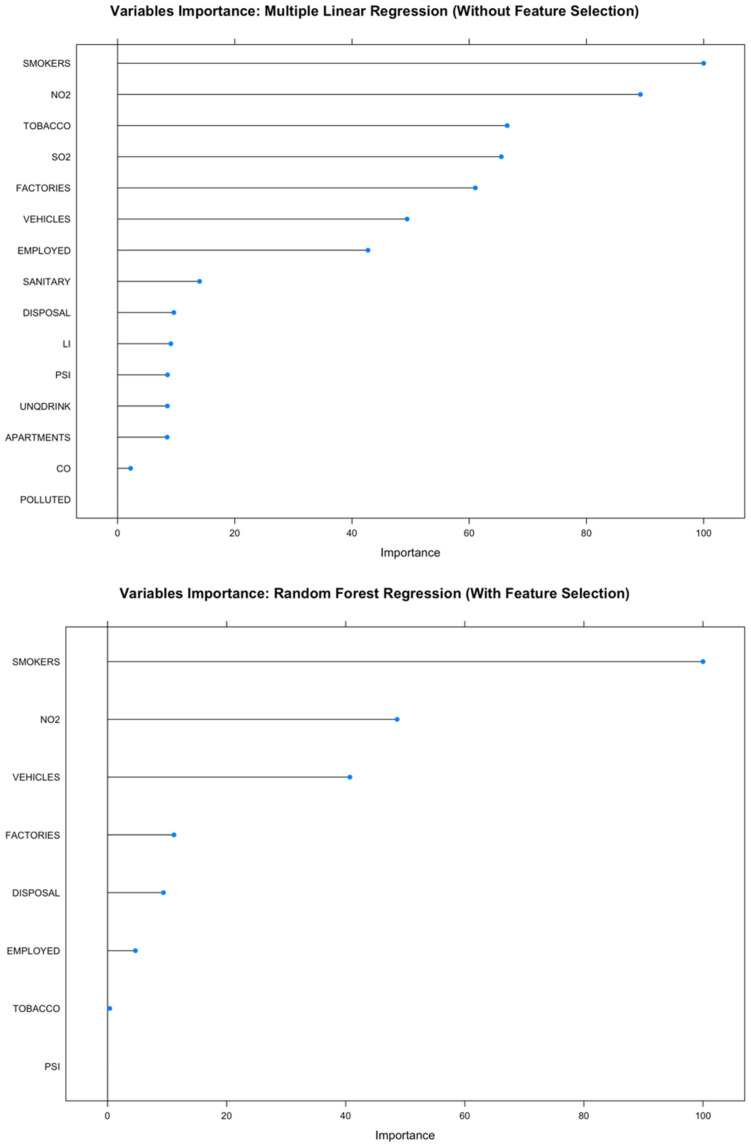
Variable importance of the random forest models.

In addition, the random forest model without feature selection and the random forest model with feature selection, which were also considered among the best models in this study, could therefore be interpreted to support the interpretation of the most contributing variables. Visualization of variable importance of features used in the random forest model without feature selection and the random forest model with feature selection is shown in Figure 5.

Figure 5 indicates that the percentage of smokers is the most important variable for both the random forest model with feature selection and the random forest model without feature selection. This supported the claim that smoking is the most important risk factor for lung cancer, and other data supporting this association are also very convincing [68,69]. It could be concluded that the random forest model with feature selection and the random forest model without feature selection were quite reliable at predicting the lung cancer incidence rate in this study.

Furthermore, the proposed predictive model can facilitate researchers and experts when analyzing and estimating lung cancer incidence rates to enable the development of more effective preventative measures. The model enables an overview of how lung cancer incidence has changed over time in a population from a variety of aspects. Medical experts, such as pharmaceutical and biotech companies, rely on incidence rates when they apply to the Food and Drug Administration (FDA) for permission to commercialize the drugs; therefore, the model can assist them in anticipating future incidents and making appropriate plans. Further, the predicted outcomes can be utilized to raise public awareness about lung cancer and how to prevent it.

## 4. Conclusions

### 4.1. Summary

The high incidence of lung cancer as one of the deadliest diseases in the world means that lung cancer needs more attention. The difficulty in obtaining complete and reliable medical data, including lung cancer data, prompted us to build predictive models of the lung cancer incidence rate. In this study, we built models to predict the lung cancer incidence rate in Taiwan using linear regression, support vector regression, random forest, K-nearest neighbor, and cubist model tree. Various risk factors were also considered when building the prediction models, such as air pollution, tobacco use, socioeconomic status, employment status, marital status, and living environment. The consideration was that historical medical data normally used for forecasting, including the lung cancer incidence rate data, are very difficult to obtain and expensive. Feature selection based on the VIF value was performed to eliminate highly correlated variables, and fivefold cross-validation was applied to evaluate the prediction model. The results showed that all models, except the linear regression model without feature selection, fit the observed data well. The cubist model tree with feature selection, which had a fairly constant performance at each fold, was the best model with the lowest RMSE and the highest R-squared, followed by the random forest model without feature selection and the random forest model with feature selection. Through these predictive models, we also found that apart from smoking, the average NO_2_ concentration, percentage of employed, and number of factories were also important factors that had a significant impact on the incidence of lung cancer in Taiwan. Therefore, reducing the risk of these factors in order to reduce the incidence of lung cancer is an urgent issue.

### 4.2. Limitation

For future research, it is suggested that researchers consider other risk factors of lung cancer, such as secondhand smoke; dietary habits; and exposure to radon, asbestos, or other cancer-causing agents. Several chronic diseases related to lung cancer are also suggested to be considered. Analyzing other types of cancer can also be a challenge for researchers. Future research may implement more machine learning algorithms or deep learning algorithms to build predictive models of the incidence rate of lung cancer or other types of cancer. The proposed model was from the whole-country viewpoint. This is a limitation if we want to use this calculation for one hospital or one country region. A regional modeling approach is recommended if the local features and data are available.

## Figures and Tables

**Table 1:** Description of the predictive model variables.

Factor	Variable (Notation)	Description	Data Type
Air pollution	Carbon monoxide (CO)	Average CO concentration (ppm)	Continuous
	2. Nitrogen dioxide (NO_2_)	Average NO_2_ concentration (ppb)	Continuous
	3. Sulfur dioxide (SO_2_)	Average SO_2_ concentration (ppb)	Continuous
	4. Ozone (O_3_)	Average O_3_ concentration (ppb)	Continuous
	5. Particulate matter 10 (PM10)	Average PM_10_ concentration (μg/m^3^)	Continuous
	6. Registered vehicles (VEHICLES)	Total number of registered vehicles, including buses, heavy trucks, sedans, light trucks, specially constructed vehicles, and motorcycles.	Discrete
	7. Factories (FACTORIES)	Total number of factories	Discrete
Tobacco use	8. Tobacco consumption per capita (TOBACCO)	Consumption of tobacco per capita aged 18 and over (pieces/year)	Discrete
	9. Smokers rate (SMOKERS)	Percentage of smokers from population aged 18 and over	Continuous
Socioeconomic status	10. Rate of low-income persons (LI)	Percentage of low-income persons from total population	Continuous
Employment status	11. Percent employed (EMPLOYED)	Percentage of employed from civilian population aged 15 and over	Continuous
	12. Unemployment rate (UNEMPLOYMENT)	Total unemployment rate	Continuous
Marital status	13. Divorce status (DIVORCE)	Divorce status of population aged 15 and over	Continuous
Living environment	14. Rate of one-story buildings (ONE)	Number of households living in one-story buildings	Continuous
	15. Rate of apartments six stories or over (APARTMENTS)	Number of households living in apartments six stories or over	Continuous
	16. Rate of days with PSI > 100 (PSI)	Percentage of days measured with PSI > 100	Continuous
	17. Availability rate of public sanitary sewers (SANITARY)	Percentage of public sanitary sewer availability	Continuous
	18. Rate of heavily polluted sections (POLLUTED)	Percentage of heavily polluted sections in the total length of major rivers	Continuous
	19. Rate of unqualified drinking water (UNQDRINK)	Percentage of unqualified drinking water as tested	Continuous
	20. Rate of proper refuse disposal (DISPOSAL)	Percentage of proper refuse disposal	Continuous
Dependent variable	21. Lung cancer incidence rate (LC)	Trachea, bronchus, and lung cancer (C33–C34) incidence rates per 100,000 in Taiwan	Continuous

**Table 2:** Selected variables from the stepwise regression and feature selection.

Factor	Predictor Variable	Stepwise Regression	Feature Selection Based on the VIF Value
Air pollution	CO	V	
	NO_2_	V	V
	SO_2_	V	
	O_3_		
	PM_10_		
	VEHICLES	V	V
	FACTORIES	V	V
Tobacco use	TOBACCO	V	V
	SMOKERS	V	V
Socioeconomic status	LI	V	
Employment status	EMPLOYED	V	V
	UNEMPLOYMENT		
Marital status	DIVORCE		
Living environment	ONE		
	APARTMENTS	V	
	PSI	V	V
	SANITARY	V	
	POLLUTED	V	
	UNQDRINK	V	
	DISPOSAL	V	V
Total number of variables		15	8

**Table 3:** Performance results of the machine learning models.

Algorithm	Fold	Without Feature Selection		With Feature Selection	
		RMSE	R-Squared	RMSE	R-Squared
Linear regression	1	17.612	0.632	22.122	0.682
	2	2.341	0.980	5.279	0.875
	3	134.232	0.532	24.519	0.827
	4	13.419	0.080	6.846	0.960
	5	4.911	0.849	10.789	0.374
	Average	34.503	0.615	13.911	0.743
Support vector regression	1	2.144	0.971	1.617	0.994
	2	3.712	0.978	5.296	0.919
	3	2.447	0.996	5.223	0.941
	4	4.055	0.922	4.244	0.984
	5	9.489	0.173	9.758	0.182
	Average	4.369	0.808	5.228	0.804
Random forest	1	5.402	0.853	4.532	0.885
	2	4.599	0.905	5.067	0.895
	3	1.732	0.969	2.448	0.935
	4	5.086	0.897	4.996	0.885
	5	7.365	0.853	7.570	0.868
	Average	4.837	0.895	4.922	0.894
K-nearest neighbor	1	2.562	0.946	7.215	0.974
	2	6.008	0.749	6.008	0.842
	3	3.925	0.875	3.516	0.923
	4	4.282	0.913	6.862	0.669
	5	10.792	0.590	6.393	0.660
	Average	5.514	0.814	5.999	0.814
Cubist model tree	1	5.817	0.831	6.524	0.853
	2	3.508	0.910	2.712	0.971
	3	5.615	0.869	2.607	0.988
	4	7.451	0.550	2.897	0.998
	5	2.007	0.987	1.808	0.990
	Average	4.880	0.829	3.310	0.960

## Data Availability

The datasets analyzed during the current study are available from the corresponding author on reasonable request.

## Appendix A

*Linear regression*: It is one of the most popular and frequently used data processing methods [70]. This method aims to identify the close cause-and-effect relationships that occur between variables and can be used to make predictions. One of the advantages of this method is that it is quite simple and easy to understand but still produces powerful insights. In practice, the most intriguing situations contain numerous predictors and a single dependent variable, necessitating the estimation of a multiple linear regression model. It is a statistical approach that models a dependent variable (*Y*) as a function of more than one independent variable (*X*_1_, *X*_2_, *X*_3_, …, *X_n_*). The multiple linear regression equation is written as $Y=f\left(X_{1},X_{2},X_{3},\ldots ,X_{n}\right)=\beta_{0}+\beta_{1}X_{1}+\beta_{2}X_{2}+\beta_{3}X_{3}+\ldots +\beta_{n}X_{n}+\varepsilon$ [50]. β_0_ is the intercept and the other β_i_’s are the slope terms associated with the corresponding independent variables (i.e., the X_i_’s). The population error term (ε) in this model is defined as the difference between the actual Y and the one predicted by the regression model (Ŷ).

*Support vector regression*: It is one of the most widely used machine learning methods for predictive problems in the medical field, such as incidence rate and survivability [39]. SVR is a method for regression cases that was developed from a popular machine learning method that has been used to solve classification cases, namely, the support vector machine (SVM), which was first identified in 1992 by Vladimir Vapnik and his colleagues [71]. The function of SVR is to discover a function as a hyperplane (separation line) in the form of a regression function that fits all input data with an error and makes it as thin as possible [72]. SVR minimizes the error by maximizing the margin of the hyperplane, it creates subclasses from the training data (support vectors); and tries to minimize the distance between the observed data and predicted data in order to improve the performance [15]. In this study, the support vector machine with a radial basis function (RBF) kernel was used. The cost parameter and the RBF kernel parameter sigma were tuned in this model.

*K-nearest neighbor (KNN)*: It is a machine learning algorithm that is called a lazy learning algorithm because of its cheap processing costs and ease of use [73]. Because of its simplicity and tolerance for high-dimensional and imperfect data, the KNN method is one of the most widely used machine learning algorithms [74,75]. This algorithm is based solely on the idea that objects that are close to each other will have similar characteristics; therefore, if the characteristic features of one of the objects are known, then its nearest neighbors can also be predicted [76]. KNN is a non-parametric method for classification or regression, with the input consisting of the k closest training instances in the feature space, but the output varies depending on whether the method is used for classification or regression. KNN saves the complete training dataset and searches it to discover k data points in the training set that are the most comparable to the data point to be categorized for generating predictions. As a result, there is no model other than the raw training dataset and the sole computation is querying this dataset [77]. In KNN regression, the response value is determined as the weighted sum of all k neighbors’ replies, with the weight being inversely proportional to the distance from the input record. The Euclidean distance is the name for this measurement. Regarding the R terms in this model, *k* was tested at {2:10} using the Caret package.

*Random forest*: It is a machine learning algorithm that is commonly used to deal with classification and regression problems because of its ease of use and flexibility [53]. This algorithm is able to produce better predictor performance compared with traditional regression or other statistical procedures, while it also protects against overfitting and detects interactions between predictors [37]. The random forest method is an extension of the bagging method as it uses both bagging and randomness features. It trains each tree with a random sample of the main dataset using row sampling and feature sampling with replacement to create a forest of uncorrelated decision trees. Basically, the random forest method combines the outputs of several decision trees, each with high variance, to achieve a single result with low variance [78]. A weak relationship between residuals and small error trees are both required for accurate regression forests. For further details about the random forest method, please refer to Breiman [79]. This study used the “rf” method from the Caret package that tunes over the mtry parameter (the number of variables picked at random in each split).

*Cubist model tree*: It is an extension of Quinlan’s M5 model tree in which corrections are added to the training set based on the nearest neighbors [80]. This powerful decision tree learner is used to generate rule-based models that can produce accurate and clear predictions in regression tasks. The cubist model generally gives better results than basic approaches, such as multivariate linear regression, and the results given are also easy to understand [81]. The balance between interpretive ability and predictive power offered by the cubist model motivated us to consider this model for this study. The cubist model was shown to be more promising in terms of deciphering complicated interactions between variables and is superior in terms of execution time [15,82]. In this model, a tree is grown, where the branches can be thought of as an “if-then” set of rules, and the terminal leaves contain predictive linear regression models. Each branch of the tree has its intermediate linear model, where these models are based on the predictors used in the previous split. A linear regression prediction model at the tree terminal node will be smoothed by considering the predictions from the linear model at the previous tree node (this happens recursively up the tree as well). Rules in the tree are pruned and/or merged for simplicity, which prefers pathways from the top to the bottom of the tree. For further details on the cubist model, please refer to Kuhn et al. [83]. The cubist model incorporates boosting via training committees (typically more than one), which is analogous to the boosting approach of successively growing trees with altered weights [82]. In the cubist model, the number of neighbors is used to modify the rule-based prediction [84]. In this study, the term *committees* was tested at 10 and the term *neighbors* was tested at {0:9} using the Caret package.
